# Pre–Post Motor–Cognitive and Shooting Performance Patterns in Security-Force Applicants During a Fixed-Order Acute-Load Protocol: A Descriptive Pilot Study

**DOI:** 10.3390/jfmk11020183

**Published:** 2026-04-30

**Authors:** Kristína Němá, Peter Kačúr, Tomáš Kozák, Ján Pohlod, Pavel Ružbarský

**Affiliations:** 1 Department of Educology of Sports, Faculty of Sport, University of Prešov, 080 01 Prešov, Slovakia; kristina.nema@unipo.sk (K.N.); pohlodj@gmail.com (J.P.); 2 Department of Sports Educology and Humanities, Faculty of Sport, University of Prešov, 080 01 Prešov, Slovakia; 3 Department of Sports Kinantropology, Faculty of Sport, University of Prešov, 080 01 Prešov, Slovakia; tomas.kotak@smail.unipo.sk (T.K.); pavel.ruzbarsky@unipo.sk (P.R.)

**Keywords:** acute physical load, shooting performance, motor–cognitive performance, Hawk Eye, security-force applicants, first-shot accuracy, spatial error distribution, descriptive pilot study

## Abstract

**Background:** Operational performance in security-force settings depends on maintaining accurate motor–cognitive and shooting performance under acute physical strain. This descriptive pilot study examined pre–post performance patterns during a fixed-order acute-load protocol and explored whether trial-level and spatial analyses identified changes beyond aggregate scores. **Methods:** Nineteen applicants (10 men, 9 women; 21.6 ± 1.0 years) completed two testing sequences separated by one week. All participants completed Sequence 1 first and Sequence 2 second; therefore, sequence-related observations were interpreted descriptively rather than causally. In both sequences, participants performed Hawk Eye testing, IPSC-based shooting, and the Jaciak Motor Coordination Test, with the order of Hawk Eye and shooting reversed between sequences. Primary outcomes were first-shot hit rate and Hawk Eye error count. Secondary and exploratory outcomes included shooting miss rate and time, Hawk Eye stimulus time, minimum and maximum response times, trial-level timing, spatial distributions, and cross-task coupling. **Results:** Heart rate increased markedly after the Jaciak test in both sequences, with end-of-test values corresponding to approximately 86–88% of age-predicted HRmax. Model-based analysis indicated lower post-load odds of a first-shot hit compared with pre-load performance. In contrast, no detectable pre–post change was observed for Hawk Eye error probability. Descriptively, first-shot hit rate decreased in Sequence 1 (62.1 ± 19.9% vs. 42.1 ± 28.2%; *p* = 0.029), while the decrease in Sequence 2 was smaller and not statistically significant (61.1 ± 24.5% vs. 52.6 ± 28.4%; *p* = 0.267). Hawk Eye error count showed no statistically detectable pre–post change in either sequence, although maximum response time decreased in Sequence 1 (*p* = 0.008). Trial-level and spatial analyses indicated additional temporal and location-specific patterns, but exploratory cross-task spatial associations were inconsistent. **Conclusions:** In this fixed-order descriptive pilot study, post-load testing was associated with lower first-shot shooting performance in this sample, whereas no statistically detectable deterioration was observed for Hawk Eye error probability. However, because the design lacked a no-load control condition and all participants completed the same sequence order, the observed pre-to-post differences cannot be attributed specifically to acute physical load. They should be interpreted as descriptive performance patterns within the implemented protocol.

## 1. Introduction

Operational performance in security force personnel relies on the integration of physical, cognitive, and motor systems that must operate effectively under conditions of acute physiological stress. Military and law enforcement officers are routinely subjected to situations that demand rapid and accurate decision-making, as well as immediate motor responses. Shooting accuracy is a key factor in professional performance and depends on efficient processing of visual information, selective attention, executive control, quick decision-making, and optimal reaction time. Previous research has shown a positive link between cognitive functioning and shooting accuracy [1,2], with more efficient information processing connected to better operational performance [3,4]. Conversely, increased cognitive load or concurrent performance of secondary tasks has been shown to impair both shooting and cognitive functions, indicating cognitive–motor interference [5,6]. Therefore, shooting performance can be viewed as a complex cognitive–motor task that is vulnerable to stress-induced changes.

Acute physical load is one of the most common stressors in security force operations. It causes significant physiological responses, including increased heart rate, metabolic shifts, and heightened arousal, which may impact motor stability and decision-making. Exercise-induced fatigue can decrease force production and cause tremors, possibly impairing weapon stability and shooting accuracy. However, empirical results are mixed. Some studies show impaired decision-making and longer shooting initiation times under combined physical and psychological stress [7]. Conversely, others find that shooting accuracy remains intact even after exhaustive exercise, especially among experienced professionals [8,9]. These differences imply that skill automatisation and individual readiness might influence how physical load affects shooting performance. Less is known, however, about how these effects occur in applicants to security forces who have yet to stabilise their cognitive–motor strategies fully.

Cognitive functions are sensitive to fluctuations in physiological arousal and fatigue. Several studies have documented impairments in short-term memory, executive functions, and psychomotor reaction time immediately after high-intensity physical exertion [10,11]. In contrast, other findings suggest a non-linear relationship between arousal and cognitive performance, whereby trained individuals may demonstrate preserved or even slightly improved aspects of attention and executive functioning following short-term intense stress [12]. Despite these findings, the relationship between cognitive functioning and shooting accuracy remains insufficiently understood immediately after acute physical load, particularly among applicants to security forces. Examining these changes may provide a more comprehensive assessment of functional readiness under conditions of physiological stress.

The present descriptive pilot study examined pre–post patterns in shooting and motor–cognitive performance in security-force applicants around an acute-load task across two fixed-order testing sequences with reversed task order. In addition to aggregate performance outcomes, the study explored whether pre–post patterns were also reflected in trial-level timing, spatially distributed errors or misses, and selected cross-task spatial correspondences. Because the sequences were not randomised or counterbalanced and no no-load control condition was included, all pre- and post- and sequence-related observations were interpreted as descriptive patterns within the implemented protocol rather than as causal evidence of acute-load effects.

## 2. Materials and Methods

### 2.1. Participants

Twenty-three applicants for security forces took part in the first sequence. Four participants were unavailable for the second sequence due to scheduling conflicts. Participants were recruited through convenience sampling from applicants undergoing the regular selection and preparatory process for security-force service during the study period. Inclusion criteria included current applicant status, age of 18 years or older, ability to complete both testing sequences, and provision of written informed consent. Exclusion criteria included any acute musculoskeletal injury, current illness, or other conditions that could compromise safe task execution or affect the validity of performance testing. The final analytical sample comprised 19 applicants for the security forces, 10 men and 9 women. The mean age was 21.6 ± 1.0 years, the mean body height was 172.6 ± 10.2 cm, the mean body mass was 67.3 ± 14.9 kg, the mean body fat percentage was 16.1 ± 6.6%, and the mean BMI was 22.3 ± 2.9 kg/m^2^. The sample size was determined pragmatically based on the availability of eligible applicants during the testing period, rather than through a formal a priori power calculation. In addition to the regular selection and preparatory process, all participants had completed a shared 13-week IPSC-based shooting preparation module within the Shooting Preparation course before experimental testing. This provided a common preparation background in IPSC shooting principles, safety procedures, shooting distance, target configuration, and target engagement requirements. However, additional individual shooting experience outside this shared course, detailed physical-training background, and baseline cognitive characteristics were not systematically quantified; therefore, these factors could not be included as moderators and are acknowledged as limitations. Because the final analytic sample was defined by cohort availability and completion of both sequences, subgroup analyses and targeted exploratory cross-task coupling analyses were interpreted cautiously and regarded as hypothesis-generating rather than confirmatory.

### 2.2. Study Design

The study used a fixed-order within-subject repeated-measures design across two testing sequences separated by a one-week interval. All participants completed Sequence 1 during the first testing session and Sequence 2 during the second testing session; sequence order was not randomised or counterbalanced. Both sequences used the same acute-load protocol, with shooting performance and motor–cognitive performance assessed before and after the Jaciak Motor Coordination Test. Accordingly, comparisons between sequences cannot separate task order, visit order, repeated exposure, familiarisation, recovery time, and post-load timing. Sequence-related observations are therefore interpreted descriptively rather than as causal timing effects.

Sequence 1: Hawk Eye test (pre-load) → shooting task (Series 1) → Jaciak Motor Coordination Test → Hawk Eye re-test (post-load) → shooting re-test (Series 2).

Sequence 2: shooting task (Series 1) → Hawk Eye test (pre-load) → Jaciak Motor Coordination Test → shooting re-test (Series 2) → Hawk Eye re-test (post-load).

Thus, the two sequences varied not only in task order but also in the interval between the acute-load stimulus and the subsequent post-load assessment of shooting and Hawk Eye performance.

### 2.3. Procedures

Participants were instructed to avoid vigorous physical activity for at least 24 h before testing to minimise the impact of residual fatigue on performance outcomes.

At the start of the initial testing session, body composition was measured using bioelectrical impedance analysis (BIA) with the InBody 230 analyser (Biospace Co., Ltd., Seoul, Republic of Korea). The assessment included body mass (BM), body height (BH), and body fat percentage (BFP) to characterise the sample.

Before the first experimental shooting task in each testing sequence, participants completed the same standardised warm-up and familiarisation procedure specific to the IPSC shooting task. This warm-up was performed before both Sequence 1 and Sequence 2. The familiarisation phase allowed participants to adjust to the shooting distance and target configuration, thereby reducing calibration behaviour during the experimental trials.

Subsequently, participants completed the Hawk Eye test, the IPSC shooting task, the Jaciak motor coordination test, and heart rate monitoring in the predefined sequence described above. Detailed descriptions of each test are provided below.

#### 2.3.1. Hawk Eye Test

Motor–cognitive performance was evaluated using the Hawk Eye protocol on the Witty·SEM system (Microgate S.r.l., Bolzano, Italy). The Witty·SEM system includes wireless, programmable LED units with proximity sensors, allowing standardised visual stimulus presentation and automated recording of response times. 

**Figure 1:**
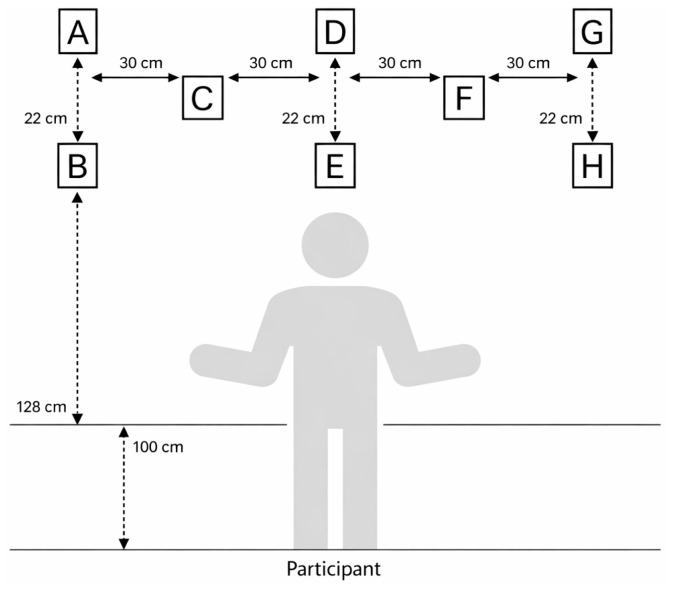
Schematic layout of Witty·SEM–Hawk Eye test.

Eight Witty·SEM units were positioned at fixed, standardised locations (A–H) in front of the participant (Figure 1). The layout comprised three upper units (A, D, and G), three lower units (B, E, and H) placed vertically below them, and two intermediate units (C and F) situated between the upper columns. The horizontal distance between adjacent upper and intermediate units was 30 cm, while the vertical distance between the upper and lower units within each column was 22 cm. The participant stood behind a marked standing line; the lower row of units was positioned 100 cm in front of this line, and the upper row 128 cm in front of it. Stimulus presentation was pseudo-randomised across the eight units, although the spatial layout remained constant for all participants and both sequences. Participants began from a predefined standing position facing the visual array and responded to each illuminated unit with a standardised upper-limb reaching action towards the active target. Each Hawk Eye block consisted of 10 trials, with medium-level stimulus presentation pseudo-randomised across the eight fixed locations (A–H).

The standardised Witty protocol controlled stimulus timing, and difficulty was adjusted automatically according to performance in previous trials. The Hawk Eye protocol was used as an integrated motor–cognitive task designed to assess visual processing speed, response timing, and response accuracy under standardised conditions.

The following block-level variables were recorded: error count, stimulus time, minimum response time, and maximum response time. The trial-level timing metric (trial_metric_raw) represented the device-recorded response time for each Hawk Eye trial, expressed in milliseconds, from visual stimulus presentation to sensor-detected participant response. Trial-level values were retained when the device recorded a valid response time, and both correct and error trials were included in the trial-level timing analysis. In addition to summary outcomes, trial-level data and spatial error distributions were stored for exploratory analyses. All Hawk Eye assessments were conducted under identical device settings in both sequences by the same examiner or research team. Participants maintained the same standing position and response mode across sequences, and no modifications to the spatial layout or task configuration were permitted between pre-load and post-load assessments.

#### 2.3.2. IPSC Shooting

Shooting performance was evaluated using a standardised protocol based on IPSC (International Practical Shooting Confederation) principles. Targets included steel IPSC Mini Popper plates, which are self-resetting reactive steel targets commonly employed in practical shooting disciplines.

**Figure 2:**
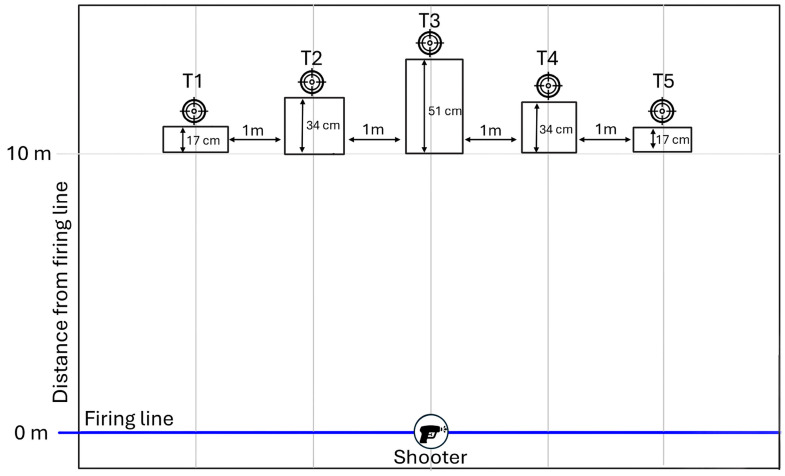
Schematic layout of the IPSC-based shooting task.

Participants engaged with five individual Mini Popper steel targets placed 10 m from the firing line. Targets were arranged horizontally with 1 m centre-to-centre spacing and mounted at varying heights (17–34–51–34–17 cm from left to right) to increase visuomotor and postural demands (Figure 2).

Each participant received 10 rounds of ammunition per trial. They were instructed to engage the targets as swiftly and accurately as possible, in accordance with IPSC safety regulations. The following performance variables were recorded: (a) first-shot hit for each target; (b) total number of successful hits; (c) number of shots per target; (d) target miss; and (e) total shooting time (s), measured from the start signal to the final shot using an electronic shot timer. The shooting task was performed twice within each experimental sequence: Series 1 before the acute-load task and Series 2 after the acute-load task.

#### 2.3.3. The Jaciak Motor Coordination Test

The Jaciak Motor Coordination Test was used to induce acute physical load. In the present study, it was not treated as a primary motor-coordination outcome; instead, it served as a short, standardised, whole-body repeated-transition task before post-load performance assessment. The test required participants to repeatedly perform a standardised sequence of four body positions over 2 min: (a) supine position with arms at sides (starting position), with heels and shoulder blades touching the floor; (b) standing upright with arms at sides; (c) prone position with arms at sides, with the chest touching the ground; and (d) standing upright with arms at sides.

Participants started in the supine position. Upon the examiner’s instruction, they performed the full sequence in the prescribed order (1 → 2 → 3 → 4) repeatedly for 2 min. Only correctly executed positions were counted. The total score was determined by the number of correctly completed positions within the time limit. All Jaciak assessments were conducted using the same verbal instructions and scoring criteria, and only correctly executed positions were included.

The test has shown acceptable concurrent validity (r ≈ 0.48–0.78) and high inter-rater reliability (ICC ≈ 0.99), with moderate test–retest reliability (ICC ≈ 0.79) [13]. Although the test is originally described as a motor-coordination assessment, it was selected here because its repeated supine–standing–prone–standing transitions provide a short, standardised, reproducible whole-body task that can be administered uniformly immediately before post-load performance assessment without additional ergometric equipment. No ratings of perceived exertion, lactate, oxygen uptake, or independent graded exercise test were collected, so physiological load characterisation was limited to end-of-test heart rate response contextualised relative to age-predicted HRmax.

#### 2.3.4. Heart Rate

Heart rate (HR) responses were monitored using the Polar Team Pro system (Polar Electro Inc., New York, USA), a chest-strap telemetry-based monitoring device. The initial HR value was obtained during testing and is therefore referred to as pre-sequence HR rather than resting HR. Subsequently, HR was recorded at the end of each task, following the order of the experimental sequence (after shooting, Hawk Eye test, Jaciak test, and repeated tasks). Heart rate served as an objective indicator of internal load, given its well-established relationship with exercise intensity and cardiovascular strain. Monitoring HR ensured that the acute-load stimulus elicited a measurable physiological response and allowed comparison of internal load across sequences. Using the sample mean age (21.6 years), the age-predicted maximal heart rate was approximately 198.4 bpm (220–age). The manipulation-check analyses reported in the Results were based on pre-sequence HR and end-of-test HR recorded immediately after the Jaciak Motor Coordination Test; these end-of-test values were also expressed descriptively as a percentage of age-predicted HRmax to contextualise relative exercise intensity, while recognising that they are not true peak HR values.

#### 2.3.5. Data Processing and Derived Variables

Raw anthropometric, shooting, and Hawk Eye records were cleaned and harmonised using a unique participant identifier. Hawk Eye and shooting data were reshaped into long format for repeated-measures analyses. For shooting, the first-shot hit was coded as 1 if the target was hit with the first shot and 0 otherwise; a target miss was coded as 1 if the target was not successfully hit within the available attempts. For Hawk Eye, error_trial was coded as 1 for an erroneous trial response and 0 for a correct response.

Spatial indices were defined from the fixed task geometry before cross-task coupling analysis and were treated as exploratory. For Hawk Eye, locations A, B, and C were classified as left-side locations, D and E as middle locations, and F, G, and H as right-side locations. Locations A, B, G, and H were classified as peripheral, whereas C, D, E, and F were classified as central. The Hawk Eye left–right asymmetry index was calculated as right-side error rate minus left-side error rate, and the Hawk Eye periphery–minus–centre index was calculated as peripheral error rate minus central error rate. For shooting, target positions 1 and 2 were classified as left-side targets, target 3 as the centre target, and target positions 4 and 5 as right-side targets. Shooting left–right asymmetry was calculated as right-side miss rate minus left-side miss rate, and the shooting periphery–minus–centre index was calculated as peripheral target miss rate minus centre target miss rate.

### 2.4. Statistical Analysis

Descriptive statistics are reported as mean ± SD for continuous variables and as counts or percentages for categorical variables. All hypothesis tests were two-sided. Analyses were performed on a complete-case basis and were limited to participants who completed both fixed-order testing sequences.

Primary model-based analyses were conducted for the two a priori primary outcomes: shooting first-shot hit and Hawk Eye error. Target-level first-shot hit (0/1) was analysed using a logistic generalised estimating equation (GEE) model with participant as the clustering variable, an exchangeable working correlation structure, robust standard errors, and predictors for phase, sequence, phase × sequence interaction, and target position. Hawk Eye trial-level error (0/1) was analysed using an analogous logistic GEE model with participant clustering, robust standard errors, and predictors for phase, sequence, phase × sequence interaction, trial number, and stimulus location. Because the design was fixed-order and not counterbalanced, sequence and phase × sequence terms were used to describe patterns rather than to infer causal timing effects. Holm adjustment was applied to the two primary phase-effect tests.

Within-sequence Wilcoxon signed-rank tests were retained as descriptive paired summaries for the block-level outcomes, with the rank-based effect size r calculated as z/√n. Secondary outcomes included shooting miss rate, shooting time, Hawk Eye stimulus time, maximum response time, and minimum response time. These secondary outcomes were interpreted descriptively, with emphasis on effect sizes and pattern consistency rather than on dichotomous significance testing.

For the continuous Hawk Eye trial-level timing metric (trial_metric_raw), values were log-transformed and analysed using a Gaussian GEE model with an exchangeable correlation structure and robust standard errors. Effects from this model are presented as multiplicative ratios with 95% confidence intervals on the original scale. Sex-related analyses, spatial distributions of errors and misses, and targeted cross-task coupling analyses were treated as exploratory. Pearson and Spearman coefficients were reported for cross-task coupling to compare linear and rank-based association patterns.

Because multiple secondary and exploratory analyses were performed, their *p*-values were not used for confirmatory inference. A sensitivity calculation for a paired pre–post comparison with N = 19, α = 0.05, and 80% power indicated that the study could detect approximately medium-to-large within-subject effects (minimum detectable dz ≈ 0.68); therefore, small-to-moderate effects, interaction effects, and subgroup effects may have remained undetected. Statistical analyses were performed in Python 3.11.9 using pandas (2.2.2), SciPy (1.13.1), and statsmodels (0.14.2).

## 3. Results

### 3.1. Manipulation Check and Shooting Performance

The manipulation check confirmed that the Jaciak Motor Coordination Test elicited a substantial acute physiological response in both fixed-order testing sequences. In Sequence 1, heart rate increased from 106.1 ± 19.9 bpm at pre-sequence measurement to 169.8 ± 20.2 bpm at the end of the Jaciak test (Δ = 63.8 ± 29.7 bpm, *p* < 0.001, r = 0.85). In Sequence 2, heart rate increased from 106.8 ± 18.1 bpm to 174.9 ± 7.6 bpm at the end of the Jaciak test (Δ = 68.2 ± 17.7 bpm, *p* < 0.001, r = 0.88). Using the sample mean age (21.6 years), the age-predicted HRmax was approximately 198.4 bpm; therefore, the mean end-of-test HR after the Jaciak task corresponded to approximately 85.6% of age-predicted HRmax in Sequence 1 and 88.2% in Sequence 2. These values are reported only as contextual markers of relative exercise intensity because they represent end-of-test readings rather than true peak HR values. Mean Jaciak scores were 78.2 ± 9.5 in Sequence 1 and 81.8 ± 9.3 in Sequence 2.

The primary model-based analysis of target-level first-shot hit showed higher odds of a first-shot hit in the pre-load phase than in the post-load phase (OR = 2.28, 95% CI 1.25–4.15, *p* = 0.007; Holm-adjusted *p* = 0.014), indicating lower first-shot performance after the acute-load task. The phase × sequence interaction was not statistically significant (OR = 0.62, 95% CI 0.31–1.24, *p* = 0.178), so the apparent difference between sequences should be interpreted descriptively. For Hawk Eye trial-level error probability, the primary model showed no statistically detectable pre–post phase effect (OR = 1.22, 95% CI 0.93–1.61, *p* = 0.156; Holm-adjusted *p* = 0.156) and no phase × sequence interaction (OR = 0.96, 95% CI 0.66–1.40, *p* = 0.833).

Shooting outcomes are summarised in Table 1. The descriptive paired summaries showed that the primary shooting outcome, first-shot hit rate, decreased from pre-load to post-load in Sequence 1, from 62.1 ± 19.9% to 42.1 ± 28.2%, representing a drop of 20.0 percentage points (*p* = 0.029, r = −0.50). In Sequence 2, the first-shot hit rate also declined, but the change was smaller and not statistically significant, decreasing from 61.1 ± 24.5% to 52.6 ± 28.4% (Δ = −8.5 percentage points, *p* = 0.267, r = −0.25). Because no formal phase × sequence interaction was detected in the primary model and the sequence order was fixed, these within-sequence differences are interpreted as descriptive post-load patterns rather than evidence that one sequence caused a different effect.

Secondary shooting outcomes showed a similar overall pattern but with weaker statistical support. Shooting time did not change significantly in either sequence, increasing from 11.76 ± 3.59 s to 13.01 ± 4.06 s in Sequence 1 (*p* = 0.145, r = 0.33) and from 10.85 ± 5.14 s to 11.41 ± 5.22 s in Sequence 2 (*p* = 0.568, r = 0.13). Miss rate increased descriptively in Sequence 1 from 3.2 ± 7.5% to 14.7 ± 25.7% (*p* = 0.075, r = −0.41), while it remained essentially unchanged in Sequence 2, shifting from 9.5 ± 22.5% to 8.4 ± 19.2% (*p* = 0.891, r = −0.03). The mean attempts per hit increased numerically in both sequences, from 1.66 ± 0.40 to 1.88 ± 0.52 in Sequence 1 (*p* = 0.265, r = 0.26, n = 18) and from 1.57 ± 0.51 to 1.75 ± 0.86 in Sequence 2 (*p* = 0.775, r = −0.07, n = 19), but neither change was statistically significant.

**Figure 3:**
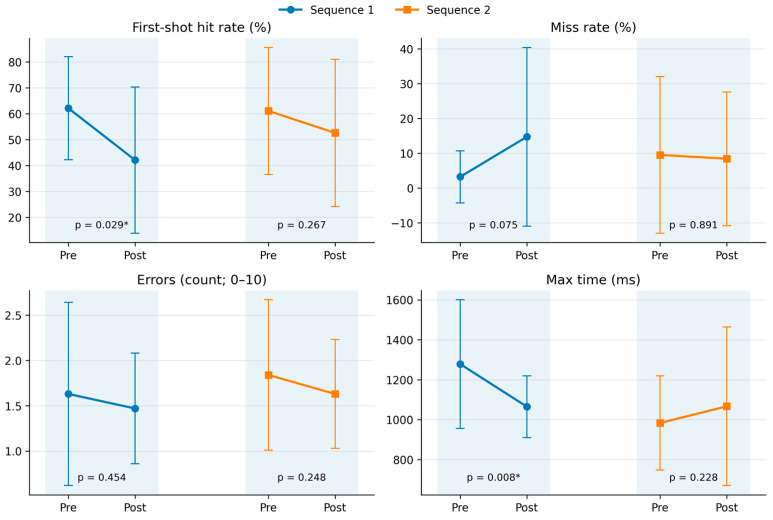
Descriptive pre-load and post-load changes in the main shooting and Hawk Eye performance outcomes across the two fixed-order testing sequences. Asterisks indicate statistically significant within-sequence pre–post differences at *p* < 0.05, based on Wilcoxon signed-rank tests.

Overall, the shooting results suggest that post-load changes were more pronounced in first-shot accuracy than in total completion time. Still, sequence-specific differences remain descriptive due to the fixed-order design (Figure 3).

### 3.2. Changes in Hawk Eye Motor–Cognitive Performance

Hawk Eye outcomes are displayed in Table 1. The primary Hawk Eye measure, error count, did not show a statistically detectable pre–post change in either sequence. In Sequence 1, error count slightly decreased from 1.63 ± 1.01 to 1.47 ± 0.61 (*p* = 0.454, r = −0.17). In Sequence 2, it decreased from 1.84 ± 0.83 to 1.63 ± 0.60 (*p* = 0.248, r = −0.26). These findings indicate that no detectable deterioration in overall Hawk Eye accuracy was observed in this sample; they should not be interpreted as proof of no effect.

Among the secondary Hawk Eye outcomes, stimulus time showed no significant pre–post change in either sequence. In Sequence 1, it decreased from 558.9 ± 304.8 ms to 455.2 ± 142.1 ms (*p* = 0.155, r = −0.33), while in Sequence 2, it decreased from 548.1 ± 246.3 ms to 471.5 ± 205.8 ms (*p* = 0.219, r = −0.28). Minimum response time also remained statistically unchanged, decreasing from 431.1 ± 169.6 ms to 378.6 ± 87.6 ms in Sequence 1 (*p* = 0.166, r = −0.32) and from 408.7 ± 150.3 ms to 380.9 ± 174.7 ms in Sequence 2 (*p* = 0.431, r = −0.18).

The only Hawk Eye summary variable that changed significantly in the descriptive paired summaries was maximum response time in Sequence 1, which decreased from 1278.6 ± 322.3 ms to 1065.2 ± 154.7 ms (*p* = 0.008, r = −0.60). In Sequence 2, maximum response time increased from 983.3 ± 235.8 ms to 1066.9 ± 397.1 ms, but this change was not statistically significant (*p* = 0.228, r = −0.28). This decrease in maximum response time should be interpreted cautiously because repeated exposure, arousal, pacing, and automatic device adjustment may have contributed to the observed pattern.

### 3.3. Trial-Level Response Dynamics

To assess more detailed temporal response behaviour within the Hawk Eye task, the log-transformed trial-level timing metric (trial_metric_raw) was analysed using a clustered repeated-measures GEE model. The pre-load phase exhibited higher values than the post-load phase, with a ratio of 1.254 (95% CI 1.127–1.396, *p* < 0.001). On the original scale, this indicates a 25.4% difference between pre- and post-load timing values.

A strong within-block practice effect was also observed. For each additional trial, the model-estimated ratio was 0.913 (95% CI 0.900–0.926, *p* < 0.001), indicating an 8.7% decrease in the timing metric per trial as the block progressed. No significant sequence effect was detected for Sequence 2 relative to Sequence 1 (ratio = 0.940, 95% CI 0.755–1.171, *p* = 0.582), and neither the pre/post × sequence interaction (ratio = 0.874, 95% CI 0.738–1.036, *p* = 0.121) nor the pre/post × sex interaction (ratio = 0.918, 95% CI 0.765–1.103, *p* = 0.363) reached significance. Likewise, no overall sex effect was found (ratio = 0.979, 95% CI 0.794–1.207, *p* = 0.843). These results indicate that trial-level response behaviour changed across phases and successive trials, but this pattern should be interpreted alongside the clear within-block practice effect.

Sex-stratified descriptive summaries were added as Supplementary Table S1. In Sequence 1, first-shot hit rate decreased from 71.1 ± 20.3% to 46.7 ± 30.0% in women and from 54.0 ± 16.5% to 38.0 ± 27.4% in men. In Sequence 2, first-shot hit rate decreased from 66.7 ± 30.0% to 57.8 ± 27.3% in women and from 56.0 ± 18.4% to 48.0 ± 30.1% in men. These sex-stratified summaries were interpreted descriptively because the study was not powered to draw subgroup inferences.

### 3.4. Spatial Distribution of Errors and Misses

**Figure 4:**
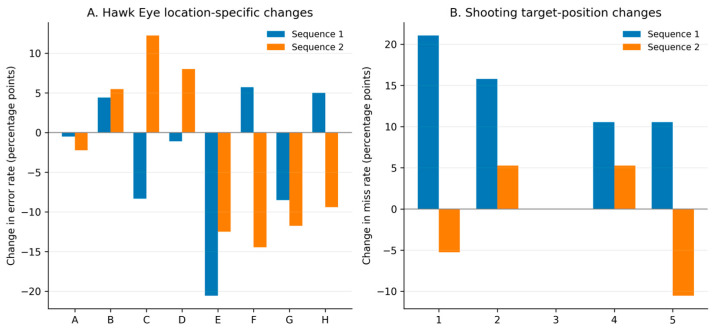
Exploratory spatial redistribution of Hawk Eye errors and shooting misses across the two fixed-order testing sequences. Note: Panel (**A**) shows location-specific changes in Hawk Eye error rates from pre-load to post-load, and Panel (**B**) shows target-position-specific changes in shooting miss rates from pre-load to post-load. Positive values indicate an increase after load, and negative values indicate a decrease.

Exploratory spatial analyses revealed that post-load changes were not evenly distributed across the Hawk Eye task layout (Figure 4). In Sequence 1, the greatest post-load increases in location-specific error rates occurred at positions F (+5.7 percentage points), H (+5.0 percentage points), and B (+4.4 percentage points). Conversely, the largest decreases occurred at E (−20.6), G (−8.5), and C (−8.3). In Sequence 2, the largest increases occurred at C (+12.2), D (+8.0), and B (+5.5), while the most notable decreases occurred at F (−14.5), E (−12.5), and G (−11.8). These values describe location-specific error redistribution and should not be interpreted as confirmatory evidence of spatial mechanisms.

A similar non-uniform descriptive pattern was evident in shooting. Target-specific miss rates did not increase equally across all targets; in Sequence 1, the largest post-load increase was observed on left-side targets (Figure 4). Overall, the spatial analyses suggest that some post-load patterns appeared in the distribution of errors and misses, even when total counts remained relatively stable.

### 3.5. Targeted Exploratory Cross-Task Coupling Analyses

Targeted exploratory cross-task coupling analyses were carried out to determine whether specific spatial indices derived from Hawk Eye performance were related to corresponding spatial indices of shooting performance (Table 2). For the periphery–minus–centre pair, the most evident signal was seen in Sequence 1, where the post–pre change in the Hawk Eye periphery–minus–centre index was positively linked to the corresponding post–pre change in shooting (Pearson r = 0.521, *p* = 0.022; Spearman ρ = 0.493, *p* = 0.032). In Sequence 2, the corresponding delta association was not significant (Pearson r = −0.213, *p* = 0.381; Spearman ρ = −0.375, *p* = 0.113).

For the left-right asymmetry pair, no significant association was observed in Sequence 1 at the pre-, post-, or delta level. In Sequence 2, no association was detected before load (Pearson r = −0.047, *p* = 0.849; Spearman ρ = −0.032, *p* = 0.898) or for the post–pre change score (Pearson r = −0.153, *p* = 0.531; Spearman ρ = −0.039, *p* = 0.873). However, after load in Sequence 2, a moderate inverse association was found between Hawk Eye and shooting left-right asymmetry (Pearson r = −0.470, *p* = 0.042; Spearman ρ = −0.484, *p* = 0.036).

## 4. Discussion

### 4.1. Main Findings

The present descriptive pilot study investigated pre–post patterns in shooting and motor–cognitive performance among security-force applicants during a fixed-order acute-load protocol. The main finding was that post-load testing under the implemented protocol was associated with lower first-shot shooting performance in the primary model. In contrast, no statistically detectable deterioration was observed for Hawk Eye error probability. Descriptive paired summaries showed a larger first-shot hit rate decrease in Sequence 1 than in Sequence 2; however, this sequence pattern cannot be interpreted causally because all participants completed the sequences in the same fixed order and no no-load control condition was included. Trial-level and spatial analyses provided additional descriptive information, but these analyses were exploratory and should be interpreted cautiously.

### 4.2. Shooting Performance in the Post-Load Context

One of the main observations of this study is the reduction in first-shot accuracy in the post-load assessment, suggesting that initial response precision may be sensitive to the post-load context. While overall shooting success may be partially preserved through corrective actions, the first response likely reflects immediate perceptual–motor processing under time constraints. Because no no-load control condition was included, this pattern cannot be attributed exclusively to acute physical load.

This finding aligns with research indicating that initial response measures may be more sensitive to stress and cognitive load than cumulative outcomes [6,7]. Studies in tactical populations further suggest that acute physical stress can decrease shooting precision and increase shot dispersion [14,15], with additional evidence showing significant declines in both accuracy and precision following fatiguing exercise [16]. These effects seem especially marked in conditions that demand postural stability.

In contrast, some studies report maintained shooting accuracy despite physical exertion, particularly among trained professionals [8,9], suggesting that experience and motor automatisation may reduce fatigue-related effects.

Taken together, these findings suggest that post-load testing within the implemented protocol may reveal vulnerability in fine motor control required for precise initial actions, even when overall task outcomes remain relatively stable. However, because the study did not include a no-load comparison condition, this pattern cannot be attributed specifically to acute physical load. These results should not be translated directly into operational performance contexts, where task completion often depends on the speed and accuracy of the first response. Nevertheless, replication in counterbalanced and controlled designs is needed before drawing firm causal conclusions.

The end-of-test HR values after the Jaciak task corresponded to approximately 85.6% and 88.2% of age-predicted HRmax in Sequence 1 and Sequence 2, respectively, indicating a marked cardiovascular response. This places the protocol in the same general high-intensity range as post-exercise pistol-shooting work, reporting an average HR of 164 bpm [8] and somewhat below the 90% HRmax uphill-run protocol used in special-forces shooting research [15]. At the same time, the Jaciak task differs from longer load-carriage protocols such as the 3-km walking test [16]. Because these protocols differ in exercise mode, duration, population, and timing of HR assessment, these comparisons are intended as contextual benchmarks rather than as direct physiological equivalents.

### 4.3. Hawk Eye Motor–Cognitive Performance in the Post-Load Context

In contrast to shooting performance, Hawk Eye motor–cognitive outcomes did not show statistically detectable deterioration in error count or error probability. This should be interpreted as an absence of detectable change in the present sample rather than as evidence that acute physical load does not affect motor–cognitive performance.

These findings align with the literature, indicating that cognitive responses to physical stress are task-dependent and non-linear [17]. While some studies report impairments in executive functions and reaction time after intense exercise [10,11], others show preserved or even improved performance under certain stress conditions [2]. Meta-analytic evidence [18] further indicates dissociated effects, with faster response times but reduced accuracy, suggesting that different cognitive aspects may respond differently to acute load.

The current findings suggest that no statistically detectable deterioration in global Hawk Eye summary outcomes was observed after the short-duration protocol in this sample. However, subtle alterations may still occur in trial dynamics and spatial distribution, and the low number of trials per block limits the reliability of fine-grained analyses.

### 4.4. Sequence-Sensitive and Trial-Level Response Patterns

The use of two testing sequences provided descriptive information about different post-load testing contexts, but it did not permit causal inference about timing effects. The decline in first-shot accuracy appeared larger in Sequence 1. Yet, the primary model did not detect a statistically significant phase × sequence interaction, and all participants completed the sequences in the same order.

Previous research indicates that the timing of physical stress relative to task execution can influence performance outcomes [19]. In the present study, however, timing, recovery, task order, and visit order were not independent. The observed pattern may also reflect arousal, repeated exposure, or familiarisation mechanisms rather than a pure load-timing effect.

Reversing task order across sequences increased ecological relevance but also introduced interpretive complexity. Because load timing, recovery processes, task sequencing, and visit order were partly confounded, the observed differences should be interpreted as fixed-order sequence-dependent patterns rather than direct causal effects.

The significant decrease in maximum Hawk Eye response time in Sequence 1 also requires caution. Although it may appear to suggest improved post-load performance, the finding is more plausibly interpreted as a combination of repeated exposure, pacing adaptation, arousal, and automatic stimulus adjustment by the device. The strong within-block practice effect in the trial-level timing model supports this cautious interpretation.

Trial-level analyses further revealed changes in within-task performance, which may reflect a combination of fatigue and short-term adaptation. Unlike global summary measures, which can smooth out subtle fluctuations, trial-level modelling seemed to detect more detailed changes in response timing, including differences between pre- and post-load phases and trends across trials.

This sensitivity to temporal dynamics aligns with evidence that physiological and cognitive responses to exercise occur across multiple timescales [18,20], which may differentially affect performance depending on when it is assessed.

These findings align with evidence suggesting that aggregate metrics might conceal important temporal dynamics [10,11]. Overall, the results emphasise the potential importance of considering both task sequence and trial-level behaviour when interpreting performance under acute physical load.

### 4.5. Spatial Redistribution of Errors and Misses

The exploratory spatial findings suggest that post-load performance patterns may be expressed not only through total accuracy but also through the spatial distribution of errors. Participants often maintained similar total performance scores, while error or miss patterns shifted across specific locations. These patterns should be interpreted as descriptive maps of task-specific performance distribution, not as clinical visual-field effects or confirmatory mechanisms.

This observation suggests that global summary metrics may not fully reflect meaningful changes in performance. Similar uneven effects of fatigue on motor control have been reported in previous studies [7,8,21].

From an applied perspective, spatial analysis may provide additional descriptive insight into how performance is organised under post-load testing conditions. Indices such as periphery–centre distribution or lateral asymmetry may complement conventional summary measures, but they require confirmation in larger samples with predefined analysis plans and multiplicity control.

### 4.6. Targeted Exploratory Cross-Task Coupling

Exploratory analyses of cross-task relationships showed that the links between motor–cognitive skills and shooting performance were inconsistent and context-dependent. Although some spatial correspondences were noted, these were not consistent across sequences.

This finding is consistent with previous research suggesting that cognitive and motor systems may react differently to acute stress and that their interaction could depend on task demands, arousal levels, and individual differences [2,7].

Furthermore, variations in recovery dynamics might explain the observed variability. Previous evidence [16,18] indicates that motor and cognitive functions may recover over different timescales. Simultaneously, factors such as tremor and neuromuscular responses [20] might further influence motor performance depending on the type and intensity of load.

Overall, these findings suggest that cognitive–motor integration under acute load is complex and context-dependent, but the present cross-task correlations are not sufficiently stable to support mechanistic conclusions. Given the exploratory nature of these analyses, the results should be interpreted cautiously and used primarily to guide future confirmatory work.

### 4.7. Methodological and Future Research Implications

The present data should not be used in isolation to define selection criteria, pass/fail standards, or training prescriptions in security-force applicants. Because the study used a fixed-order pre–post design without a no-load comparison condition, the observed first-shot decline cannot be attributed specifically to acute physical load. It may also reflect repeated exposure, visit order, familiarisation, or short-term recovery processes.

At this stage, the main contribution of the study is methodological. First-shot accuracy, trial-level timing, and spatial distribution metrics may represent useful candidate outcomes for future controlled studies because, in the present protocol, they appeared more sensitive than some aggregate scores. However, their operational value should be evaluated only after replication in designs that include a no-load control condition, counterbalancing or randomisation of task order, prespecified post-load assessment windows, and preregistered analytic plans.

### 4.8. Strengths and Limitations

This study has several strengths. The within-subject repeated-measures design minimised inter-individual variability and allowed each participant to serve as their own control. The inclusion of two specific task sequences enabled examination of performance under different post-load contexts. Furthermore, the integration of shooting performance and motor–cognitive assessment, combined with global, trial-level, and spatial indicators, provided a multi-dimensional perspective on responses to acute physical load.

Several limitations require emphasis. First, the study used a fixed-order within-subject design, and all participants completed Sequence 1 before Sequence 2. Therefore, sequence differences may reflect task order, visit order, repeated exposure, familiarisation, recovery time, or a combination of these factors. Second, no no-load control condition was included, so pre–post changes cannot be attributed exclusively to acute physical load. Third, the sample size was modest and not determined by an a priori power calculation; the sensitivity analysis indicated that only medium-to-large paired effects were likely to be detectable, limiting precision for interaction, sex-related, and cross-task analyses. Fourth, the initial HR value was obtained in the testing context and should not be interpreted as true resting HR; similarly, post-task HR values were recorded at the end of the relevant test phase and should be interpreted only as end-of-test readings. Fifth, all participants completed the same 13-week IPSC-based shooting preparation module before testing. Still, additional individual shooting experience outside this shared preparation and a detailed fitness background were not systematically quantified. Sixth, Hawk Eye blocks consisted of only 10 trials, limiting the reliability of fine-grained trial-level and spatial analyses. Seventh, although the end-of-test HR response suggested a marked cardiovascular load (approximately 86–88% of age-predicted HRmax), the Jaciak test was used as a practical acute-load stimulus rather than as a standardised fatigue protocol with comprehensive physiological measurement; RPE, lactate, oxygen uptake, and independent graded exercise testing were not collected. Finally, multiple secondary and exploratory analyses were performed; therefore, these findings should be considered hypothesis-generating.

Another important limitation is the absence of an independent baseline cognitive and perceptual-motor characterisation. Because simple reaction time, choice reaction time, working memory/executive control, and visual processing or visuospatial attention were not measured before the experimental sessions, the inter-individual variability observed in the trial-level, spatial, and targeted exploratory cross-task analyses cannot be decomposed into pre-existing participant differences, protocol-related change, or their interaction. Consequently, part of the heterogeneity observed in the exploratory analyses may reflect uncontrolled baseline cognitive/perceptual–motor differences rather than differential sensitivity to the implemented protocol. Future studies should therefore combine post-load performance testing with baseline measures of simple and choice reaction time, working memory/executive control, visual processing/visuospatial attention, and prior shooting-experience characterisation.

A controlled design capable of confirming the present pattern would need to demonstrate that the pre–post decline in first-shot accuracy is greater after an acute-load session than after an otherwise identical no-load session. Such a design should match warm-up, familiarisation, task order, and the pre- and post–interval across sessions, and ideally randomise or counterbalance the order of load and no-load visits. Inclusion of multiple post-load assessment windows would further help distinguish an immediate protocol-related response from short-term recovery. Only a statistically supported time × condition interaction in such a design would permit causal inference that acute physical load, rather than repeated exposure, visit order, or familiarisation, explains the observed change.

## 5. Conclusions

This descriptive pilot study documented pre- and post-shooting and motor–cognitive performance patterns in security-force applicants under a fixed-order acute-load protocol. In this sample, post-load testing coincided with lower first-shot shooting performance, whereas no statistically detectable change was observed for Hawk Eye error probability. Trial-level and spatial analyses provided additional descriptive information beyond aggregate scores. However, because the study lacked both counterbalancing and a no-load control condition, these patterns cannot be attributed specifically to acute physical load. Controlled randomised or counterbalanced studies with matched no-load comparison conditions are needed before these candidate markers can inform operational assessment or training practice.

## Figures and Tables

**Table 1:** Main shooting and Hawk Eye performance outcomes before and after acute physical load across the two fixed-order testing sequences.

Panel	Sequence	Outcome	Pre	Post	Wilcoxon *p*	Effect Size r	n
Shooting	Sequence 1	Shooting time (s)	11.76 ± 3.59	13.01 ± 4.06	0.145	0.33 ^†^	19
	Sequence 2	Shooting time (s)	10.85 ± 5.14	11.41 ± 5.22	0.568	0.13	19
	Sequence 1	Miss rate (%)	3.2 ± 7.5	14.7 ± 25.7	0.075	0.41 ^†^	19
	Sequence 2	Miss rate (%)	9.5 ± 22.5	8.4 ± 19.2	0.891	0.03	19
	Sequence 1	Hit rate (%)	96.8 ± 7.5	85.3 ± 25.7	0.075	0.41 ^†^	19
	Sequence 2	Hit rate (%)	90.5 ± 22.5	91.6 ± 19.2	0.891	0.03	19
	Sequence 1	First-shot hit rate (%)	62.1 ± 19.9	42.1 ± 28.2	0.029 *	0.5 ^‡^	19
	Sequence 2	First-shot hit rate (%)	61.1 ± 24.5	52.6 ± 28.4	0.267	0.25	19
	Sequence 1	Mean attempts per hit	1.66 ± 0.40	1.88 ± 0.52	0.265	0.26	18
	Sequence 2	Mean attempts per hit	1.57 ± 0.51	1.75 ± 0.86	0.775	0.07	19
Hawk Eye	Sequence 1	Errors (count; 0–10)	1.63 ± 1.01	1.47 ± 0.61	0.454	0.17	19
	Sequence 2	Errors (count; 0–10)	1.84 ± 0.83	1.63 ± 0.60	0.248	0.26	19
	Sequence 1	Stimulus time (ms)	558.9 ± 304.8	455.2 ± 142.1	0.155	0.33 ^†^	19
	Sequence 2	Stimulus time (ms)	548.1 ± 246.3	471.5 ± 205.8	0.219	0.28	19
	Sequence 1	Max time (ms)	1278.6 ± 322.3	1065.2 ± 154.7	0.008 **	0.6 ^‡^	19
	Sequence 2	Max time (ms)	983.3 ± 235.8	1066.9 ± 397.1	0.228	0.28	19
	Sequence 1	Min time (ms)	431.1 ± 169.6	378.6 ± 87.6	0.166	0.32 ^†^	19
	Sequence 2	Min time (ms)	408.7 ± 150.3	380.9 ± 174.7	0.431	0.18	19

Note: Values are presented as mean ± SD. Sequence 1 and Sequence 2 were completed in the same fixed order by all participants and should be interpreted as descriptive testing contexts rather than counterbalanced conditions. Pre–post comparisons within each sequence were tested using the Wilcoxon signed-rank test. Effect sizes are reported as rank-based r values (z/√n) and should not be interpreted as Cohen’s d. Effect size interpretation: small = 0.10–0.29, medium = 0.30–0.49, and large ≥0.50. ^†^ medium effect size; ^‡^ large effect size. * *p* < 0.05; ** *p* < 0.01.

**Table 2:** Targeted exploratory cross-task coupling analyses between selected Hawk Eye spatial indices and corresponding shooting spatial indices.

Spatial Pair	Sequence	Phase	Pearson r	Pearson *p*	Spearman ρ	Spearman *p*	n
Periphery–minus–centre change	Sequence 1	Δ (post–pre)	0.521	0.022 *	0.493	0.032 *	19
Periphery–minus–centre change	Sequence 2	Δ (post–pre)	−0.213	0.381	−0.375	0.113	19
Left-right asymmetry	Sequence 1	Pre	−0.220	0.364	−0.121	0.623	19
Left-right asymmetry	Sequence 1	Post	−0.105	0.670	−0.073	0.768	19
Left-right asymmetry	Sequence 1	Δ (post–pre)	−0.078	0.751	−0.069	0.778	19
Left-right asymmetry	Sequence 2	Pre	−0.047	0.849	−0.032	0.898	19
Left-right asymmetry	Sequence 2	Post	−0.470	0.042 *	−0.484	0.036 *	19
Left-right asymmetry	Sequence 2	Δ (post–pre)	−0.153	0.531	−0.039	0.873	19

Note: Pearson and Spearman coefficients are reported for the two targeted spatial pairs. Asterisks indicate statistically significant associations (*p* < 0.05). These unadjusted exploratory associations should be interpreted as hypothesis-generating and not as confirmatory evidence of cross-task coupling.

## Data Availability

The original data presented in this study are openly available in Zenodo at https://doi.org/10.5281/zenodo.19258081.

## Supplementary Materials

The following supporting information can be downloaded at: https://www.mdpi.com/article/10.3390/jfmk11020183/s1, Table S1: Sex-stratified descriptive pre–post changes; Table S2: Trial-level timing model output and model-based predicted means; Table S3: Location-specific Hawk Eye error rate changes and target-position-specific shooting miss rate changes.

## Abbreviations

The following abbreviations are used in this manuscript:

BFP	Body fat percentage
BH	Body height
BIA	Bioelectrical impedance analysis
BMI	Body mass index
BM	Body mass
CI	Confidence interval
GEE	Generalised estimating equation
HR	Heart rate
ICC	Intraclass correlation coefficient
IPSC	International Practical Shooting Confederation
SD	Standard deviation
