# Supplementary material for: Pre–Post Motor–Cognitive and Shooting Performance Patterns in Security-Force Applicants During a Fixed-Order Acute-Load Protocol: A Descriptive Pilot Study
Source: J Funct Morphol Kinesiol. 2026 Apr 30;11(2):183. doi: 10.3390/jfmk11020183 (PMC13214928; doi:10.3390/jfmk11020183)
Supplement: Supplementary file 1 [file jfmk-11-00183-s001.zip › jfmk-4251228-supplementary.pdf]

# Supplementary Tables

**Table S1.** Sex-stratified descriptive pre–post changes.

| Outcome | Condition | Sex | n | Pre (mean ± SD) | Post (mean ± SD) | Δ post-pre |
|---|---|---|---|---|---|---|
| WITTY Errors (0–10) | A | F | 9 | 1.67 ± 0.87 | 1.44 ± 0.73 | -0.22 ± 0.44 |
| WITTY Stimulus time (ms) | A | F | 9 | 557.1 ± 249.0 | 485.4 ± 191.9 | -71.7 ± 238.7 |
| WITTY Max time (ms) | A | F | 9 | 1229.4 ± 217.7 | 1045.9 ± 137.7 | -183.6 ± 217.7 |
| WITTY Errors (0–10) | A | M | 10 | 1.60 ± 1.17 | 1.50 ± 0.53 | -0.10 ± 1.20 |
| WITTY Stimulus time (ms) | A | M | 10 | 560.5 ± 361.5 | 428.0 ± 76.9 | -132.5 ± 344.2 |
| WITTY Max time (ms) | A | M | 10 | 1322.9 ± 401.3 | 1082.6 ± 174.1 | -240.3 ± 348.4 |
| WITTY Errors (0–10) | B | F | 9 | 1.89 ± 0.78 | 1.33 ± 0.50 | -0.56 ± 0.73 |
| WITTY Stimulus time (ms) | B | F | 9 | 632.2 ± 258.7 | 449.7 ± 154.1 | -182.6 ± 220.8 |
| WITTY Max time (ms) | B | F | 9 | 1091.8 ± 182.1 | 1156.4 ± 342.8 | 64.7 ± 194.0 |
| WITTY Errors (0–10) | B | M | 10 | 1.80 ± 0.92 | 1.90 ± 0.57 | 0.10 ± 0.74 |
| WITTY Stimulus time (ms) | B | M | 10 | 472.3 ± 219.9 | 491.2 ± 250.4 | 18.9 ± 234.8 |
| WITTY Max time (ms) | B | M | 10 | 885.6 ± 243.6 | 986.3 ± 442.4 | 100.7 ± 307.5 |
| Shooting time (s) | A | F | 9 | 12.57 ± 4.41 | 13.08 ± 4.37 | 0.50 ± 5.32 |
| Miss rate (%) | A | F | 9 | 2.2 ± 6.7 | 8.9 ± 17.6 | 6.7 ± 20.0 |
| First-shot hit rate (%) | A | F | 9 | 71.1 ± 20.3 | 46.7 ± 30.0 | -24.4 ± 31.3 |
| Shooting time (s) | A | M | 10 | 11.03 ± 2.69 | 12.94 ± 3.99 | 1.91 ± 3.09 |
| Miss rate (%) | A | M | 10 | 4.0 ± 8.4 | 20.0 ± 31.3 | 16.0 ± 35.0 |
| First-shot hit rate (%) | A | M | 10 | 54.0 ± 16.5 | 38.0 ± 27.4 | -16.0 ± 35.0 |
| Shooting time (s) | B | F | 9 | 11.60 ± 6.48 | 11.92 ± 5.89 | 0.32 ± 5.21 |
| Miss rate (%) | B | F | 9 | 15.6 ± 31.3 | 8.9 ± 20.3 | -6.7 ± 28.3 |
| First-shot hit rate (%) | B | F | 9 | 66.7 ± 30.0 | 57.8 ± 27.3 | -8.9 ± 24.7 |
| Shooting time (s) | B | M | 10 | 10.17 ± 3.81 | 10.95 ± 4.82 | 0.78 ± 3.79 |
| Miss rate (%) | B | M | 10 | 4.0 ± 8.4 | 8.0 ± 19.3 | 4.0 ± 15.8 |
| First-shot hit rate (%) | B | M | 10 | 56.0 ± 18.4 | 48.0 ± 30.1 | -8.0 ± 31.6 |

Note: These summaries are descriptive only. The study was not powered for sex-specific inference. All sex-stratified rows use the final paired sample after participant-identifier harmonisation (women n = 9; men n = 10).

**Table S2.** Trial-level Hawk Eye timing model (trial_metric_raw; log scale, ratios).

| Effect | ratio (95% CI) | % change | p |
|---|---|---|---|
| Pre vs Post (reference=Post) | 1.254 [1.127, 1.396] | 25.4% | <0.001 |
| Condition B vs A (reference=A) | 0.940 [0.755, 1.171] | -6.0% | 0.582 |
| Men vs Women (reference=Women) | 0.979 [0.794, 1.207] | -2.1% | 0.843 |
| Interaction: (Pre vs Post) × Condition B | 0.874 [0.738, 1.036] | -12.6% | 0.121 |
| Interaction: (Pre vs Post) × Men | 0.918 [0.765, 1.103] | -8.2% | 0.363 |
| Within-block trial number (per +1 trial) | 0.913 [0.900, 0.926] | -8.7% | <0.001 |

**Note:** Ratios are presented on the original scale after log-link back-transformation.

**Table S2 continued.** Model-based predicted means for trial_metric_raw by condition and sex.

| Condition | sex | Predicted mean (Pre) ms | Predicted mean (Post) ms | Δ Post-Pre (ms) | Pre/Post ratio |
|---|---|---|---|---|---|
| A | F | 828.8 | 660.8 | -168.0 | 1.254 |
| A | M | 745.4 | 647.0 | -98.4 | 1.152 |
| B | F | 681.1 | 621.2 | -59.9 | 1.096 |
| B | M | 612.5 | 608.3 | -4.2 | 1.007 |

**Note:** Predicted means are based on the trial-level GEE model.

**Table S3A.** Hawk Eye location-specific post–pre change in error rate.

| Sequence | Location | Δ error rate (pp) |
|---|---|---|
| Sequence 1 | A | -0.5 |
| Sequence 1 | B | 4.4 |
| Sequence 1 | C | -8.3 |
| Sequence 1 | D | -1.1 |
| Sequence 1 | E | -20.6 |
| Sequence 1 | F | 5.7 |
| Sequence 1 | G | -8.5 |
| Sequence 1 | H | 5.0 |
| Sequence 2 | A | -2.2 |
| Sequence 2 | B | 5.5 |
| Sequence 2 | C | 12.2 |
| Sequence 2 | D | 8.0 |
| Sequence 2 | E | -12.5 |
| Sequence 2 | F | -14.5 |
| Sequence 2 | G | -11.8 |
| Sequence 2 | H | -9.4 |

**Note:** Positive values indicate a post-load increase; negative values indicate a post-load decrease.

**Table S3B.** Shooting target-position-specific post–pre change in miss rate.

| Sequence | Target position | Δ miss rate (pp) |
|---|---|---|
| Sequence 1 | 1 | 21.1 |
| Sequence 1 | 2 | 15.8 |
| Sequence 1 | 3 | 0.0 |
| Sequence 1 | 4 | 10.5 |
| Sequence 1 | 5 | 10.5 |
| Sequence 2 | 1 | -5.3 |
| Sequence 2 | 2 | 5.3 |
| Sequence 2 | 3 | 0.0 |
| Sequence 2 | 4 | 5.3 |

| Sequence | Target position | Δ miss rate (pp) |
|---|---|---|
| Sequence 2 | 5 | -10.5 |

**Note:** Targets were engaged left to right. Positive values indicate a post-load increase; negative values indicate a post-load decrease.
